# Blood Flow Restriction Training in Stroke Rehabilitation: A Narrative Review of Current Evidence and Future Directions

**DOI:** 10.1002/brb3.71832

**Published:** 2026-10-09

**Authors:** Jiawei Tong, Lei Song, Juan Jin, Fangfang Li, Tao Huang, Hang Ying, Jiawei Zhu, Jue Wang, Jie Zhuang

**Affiliations:** ^1^ Department of Rehabilitation Medicine The Second Rehabilitation Hospital of Shanghai Shanghai China; ^2^ School of Rehabilitation Gannan Medical University Jiangxi China

**Keywords:** blood flow restriction training, functional recovery, muscle adaptation, neuroplasticity, stroke rehabilitation

## Abstract

**Background:**

Post‐stroke motor deficits cause disability, while paresis and comorbidities may limit high‐intensity training. Blood flow restriction training (BFRT) combines low‐intensity exercise with partial vascular occlusion and may promote neuromuscular adaptations at low loads.

**Methods:**

This narrative review synthesized peer‐reviewed evidence on BFRT in stroke rehabilitation. Stroke‐specific clinical studies, particularly randomized controlled trials (RCTs), were prioritized for evaluating clinical effects; experimental, preclinical, physiological, mechanistic, review, and guideline evidence provided context. RCT data included participant characteristics, protocols, outcomes, and adverse events. Two reviewers independently assessed risk of bias using the Cochrane Risk of Bias tool (RoB 1). Findings were synthesized qualitatively without meta‐analysis or GRADE assessment.

**Results:**

Sixty‐seven reports were included, comprising eight stroke‐specific clinical studies and 59 contextual reports. Participants represented early, subacute, and chronic stroke stages, although stroke stage and baseline impairment were inconsistently reported. Studies generally enrolled medically stable individuals with adequate motor control, postural stability, or walking capacity; those with severe paresis or substantial mobility dependence were underrepresented. Improvements were reported in strength, motor function, balance, walking, mobility, activities of daily living, muscle morphology, and cortical activation. However, between‐group benefits were inconsistent, and BFRT was not consistently superior to conventional, unrestricted, or high‐intensity resistance exercise. No serious BFRT‐related adverse events were reported, but safety reporting was incomplete.

**Conclusion:**

BFRT may be a useful low‐load adjunct for selected stroke survivors, particularly in lower‐limb rehabilitation. Evidence remains preliminary because of risk of bias, small samples, heterogeneous protocols, short follow‐up, and limited representation of severely impaired patients. Well‐controlled studies are needed before broad implementation.

## Graphical abstract

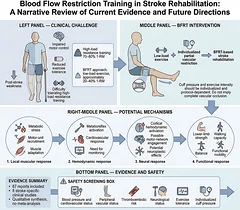

## 1 Introduction

Stroke remains a major global health burden and a leading cause of long‐term disability (GBD 2023 Causes of Death Collaborators 2025). Ischemic stroke can initiate excitotoxic, inflammatory, apoptotic, and ferroptotic processes that contribute to tissue injury and neurological impairment (Delgado‐Martín and Martínez‐Ruiz 2024; Haupt et al. 2023; Yang et al. 2025; Castillo‐González and González‐Rey 2025). Although acute interventions focused on reperfusion have improved clinical outcomes in eligible patients, their application remains limited by narrow treatment windows, contraindications, and the risk of hemorrhagic complications (Sarraj et al. 2024; Elsaid et al. 2020; Feil et al. 2020; Prandin et al. 2025; Rodríguez‐Vázquez et al. 2022; Huang et al. 2025b; Ansari et al. 2025). These limitations underscore the need to pursue targeted neuroprotective therapies and rehabilitation strategies that can be initiated after stroke and adapted to patients with residual motor deficits and medical comorbidities.

Hemiparesis is one of the most common and disabling consequences of stroke and substantially limits mobility, independence, and participation in rehabilitation (Feng et al. 2025; Qin et al. 2026; Li et al. 2025). High‐load resistance training (HLRT), typically performed at approximately 70%–80% of the one‐repetition maximum (1‐RM), can improve muscle strength and physical function. However, patients with post‐stroke weakness, spasticity, impaired motor control, cardiovascular vulnerability, or reduced exercise tolerance may be unable to safely or effectively perform high‐load resistance exercise. Low‐load training approaches may therefore provide a more feasible alternative or adjunct for selected individuals who cannot tolerate conventional resistance‐training loads. Current stroke rehabilitation guidelines also emphasize individualized, task‐oriented, and progressively dosed rehabilitation according to motor capacity and medical status (Winstein et al. 2016). More broadly, post‐stroke fitness training has been associated with improvements in physical capacity, although the effects vary according to training mode, intensity, and participant characteristics (Saunders et al. 2020).

Blood flow restriction training (BFRT) involves applying a pneumatic cuff to the proximal portion of a limb to restrict venous outflow and partially limit arterial inflow during exercise (Mannozzi et al. 2023; Huang et al. 2025a). BFRT is commonly performed with external loads of approximately 20%–40% of the 1‐RM. In healthy, athletic, and selected clinical populations, systematic reviews and meta‐analyses have reported improvements in muscle strength and hypertrophy after low‐load BFRT, although the magnitude of these adaptations varies according to the comparator, training protocol, and participant characteristics (Hughes et al. 2017; Slysz et al. 2016; Lixandrão et al. 2018; Centner et al. 2019). However, these findings do not establish equivalence with HLRT, and their applicability to individuals after stroke remains uncertain. In recent years, BFRT has also been investigated in clinical populations with musculoskeletal disorders, sarcopenia, and neurological conditions, including stroke (Cummings and Madhavan 2024; Bai et al. 2025).

Despite increasing interest in BFRT for stroke rehabilitation, the current evidence remains limited and heterogeneous. Existing studies vary substantially in participant characteristics, stroke stage, intervention protocols, cuff‐pressure selection, exercise intensity, and outcome measures. In addition, the comparative effectiveness and safety of BFRT relative to conventional rehabilitation and HLRT remain uncertain. Evidence concerning upper‐limb function, cognitive outcomes, long‐term functional recovery, and the physiological mechanisms underlying potential clinical effects is particularly sparse. These limitations make it difficult to determine which patients are most likely to benefit from BFRT and which training parameters can be applied safely and effectively. Accordingly, a comprehensive synthesis of the available clinical, mechanistic, and safety evidence is warranted.

This narrative review synthesizes the current clinical and mechanistic evidence regarding BFRT in stroke rehabilitation. Clinical outcomes, including lower‐limb strength and morphology, walking, balance, and functional mobility, are examined alongside available evidence concerning safety. Human mechanistic studies and relevant preclinical research are considered to clarify the possible local, systemic, and central responses associated with BFRT. Particular attention is given to the characteristics of the studied populations, intervention protocols, outcome measures, and methodological limitations of the available clinical studies. Finally, this review identifies important evidence gaps and discusses the research required to determine whether BFRT can be implemented more broadly in stroke rehabilitation.

## 2 Methods

This narrative review used a structured approach to literature searching, study selection, data extraction, risk‐of‐bias assessment, and qualitative synthesis. The reporting structure was informed by general principles for improving the transparency and quality of narrative reviews (Baethge et al. 2019). A structured literature search was conducted in PubMed, Scopus, Web of Science, and CNKI from database inception to June 2026. The search strategy included combinations of the following terms: “BFRT,” “BFR training,” “Kaatsu,” “stroke rehabilitation,” “hemiplegia,” and “cerebral ischemia.”

Eligibility criteria were applied to all records screened for potential inclusion in this narrative review. Reports were eligible if they addressed BFRT or exercise‐based partial vascular restriction in relation to stroke rehabilitation or post‐stroke recovery mechanisms. Eligible evidence included primary clinical studies, experimental or preclinical studies, physiological and mechanistic investigations, relevant systematic or narrative reviews, and clinical guidelines. Clinical studies were required to involve adults with a confirmed diagnosis of stroke and to evaluate BFRT or exercise‐based partial vascular restriction, alone or combined with a structured exercise or rehabilitation modality, while reporting at least one relevant clinical, functional, neurophysiological, mechanistic, or safety outcome. Experimental, mechanistic, review, and guideline evidence was included to provide contextual information on the potential mechanisms, safety, and clinical applicability of BFRT. However, claims regarding primary clinical efficacy were based only on original clinical studies involving individuals with stroke.

Records were excluded if they were unrelated to BFRT, stroke rehabilitation, post‐stroke recovery, or the mechanistic, safety, and clinical context relevant to the objectives of this review; did not provide information relevant to the clinical or contextual synthesis; or met the publication‐type exclusion criteria specified below. Duplicate or overlapping publications were removed, with the most complete report retained for the primary clinical or experimental synthesis where applicable. Conference abstracts, protocols, editorials, commentaries, letters without original data, and publications for which the full text could not be obtained after reasonable retrieval attempts were excluded. Case reports and single‐case studies were excluded from the primary clinical evidence synthesis because they provide limited evidence for treatment efficacy, although they could be considered separately if they contained clinically relevant safety information.

Two reviewers independently screened the titles and abstracts of potentially relevant records and subsequently assessed the full texts of eligible articles. Disagreements were resolved through discussion and consensus.

Detailed data extraction was performed for the included randomized controlled trials (RCTs). Extracted data included study design, sample size, stroke type, time since stroke, stroke recovery stage (e.g., acute, subacute, or chronic), baseline motor impairment, ambulatory status, intervention characteristics, including cuff pressure, exercise intensity, and training frequency, outcome measures, and reported adverse events. When stroke stage or impairment severity was not explicitly reported, the available eligibility criteria and baseline functional measures were recorded without assigning an unverified severity category. The risk of bias in the included RCTs was independently assessed by two reviewers using the Cochrane Risk of Bias tool. Disagreements were resolved through discussion and consensus.

The study‐selection process is summarized in Figure 1, which presents the PRISMA 2020 flow diagram of records identified, screened, assessed for eligibility, and included in this narrative review (Page et al. 2021). The 67 included reports comprised primary clinical studies involving individuals with stroke, experimental and preclinical studies, physiological and mechanistic investigations, relevant reviews, and clinical guidelines.

**FIGURE 1:**
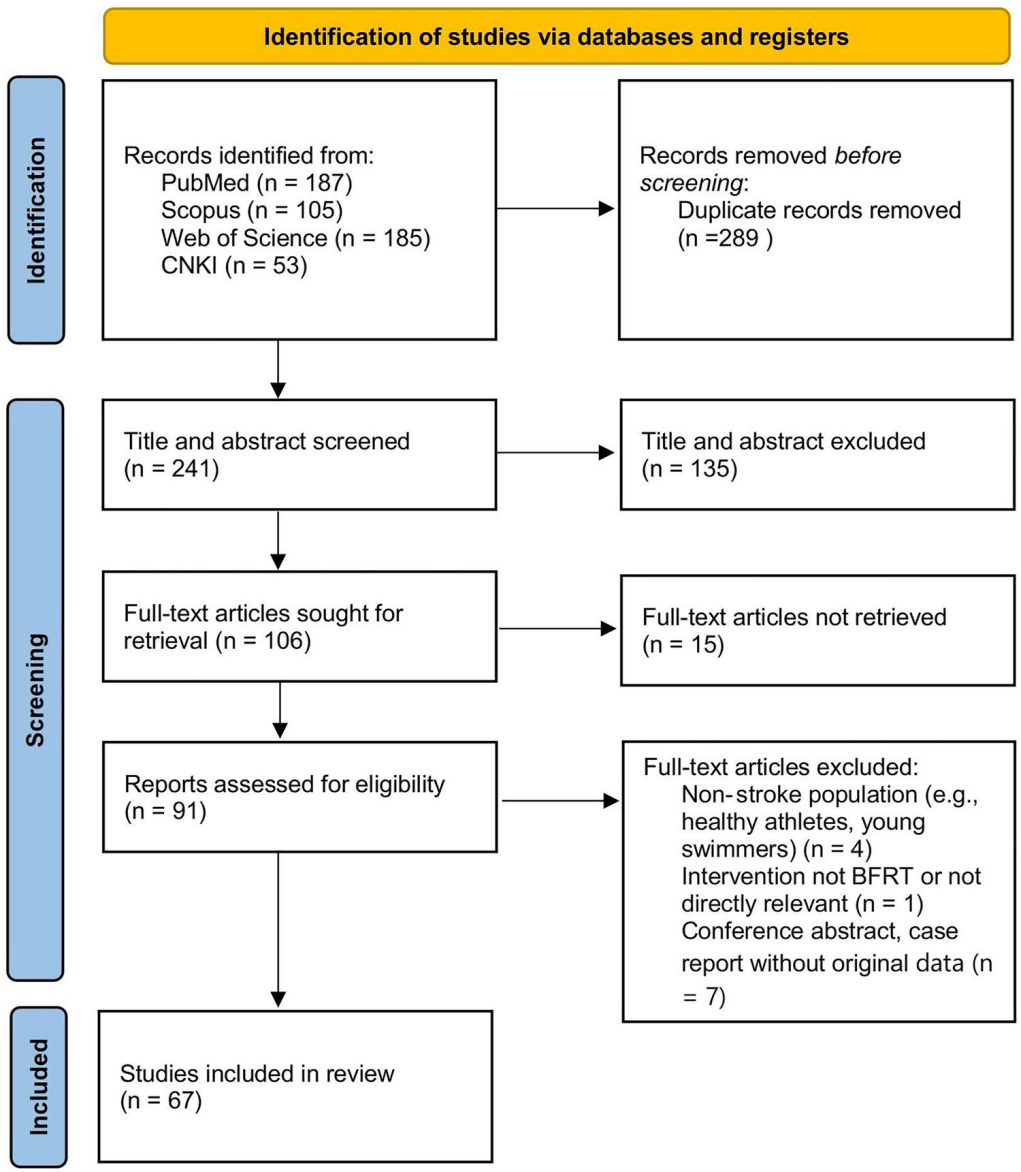
**PRISMA flow diagram of the literature search and study selection process**. Records were identified from PubMed, Scopus, Web of Science, and CNKI. After removal of duplicate records, titles and abstracts were screened, and potentially eligible reports underwent full‐text assessment. Reports were excluded because they were unrelated to BFRT, stroke rehabilitation, post‐stroke recovery, or the mechanistic, safety, and clinical context relevant to the objectives of this review; did not provide information relevant to the clinical or contextual synthesis; or represented an ineligible publication type. Finally, 67 reports were included in this narrative review.

## 3 Current Clinical Evidence for BFRT in Stroke Rehabilitation

### 3.1 Stroke Stage and Baseline Motor Impairment of Participants

The included clinical studies enrolled heterogeneous but generally selected populations of stroke survivors. Participants were recruited across different stages of stroke recovery. Several studies focused on the early recovery or subacute phase, generally enrolling participants from several weeks to 6 months after stroke (Bai et al. 2025; Li et al. 2025; Qin et al. 2026; Sun et al. 2024). Other studies included participants with longer or more variable time since stroke, including mixed subacute and chronic samples or predominantly chronic populations (Ahmed et al. 2024; Feng et al. 2023; Feng et al. 2025; Kjeldsen et al. 2022). However, definitions of stroke stage and reporting of time since stroke were not consistent across studies, limiting clear comparisons between recovery phases. Study‐specific information on time since stroke and stroke stage is summarized in Table 1.

**TABLE 1:** Characteristics and main findings of clinical studies of blood flow restriction training in stroke rehabilitation.

Author (Year)	Study design	Sample	Stroke type	Time since stroke/stroke stage	Baseline motor impairment and functional status	Intervention	Comparator	Duration	Main between‐group findings	Safety
Ahmed et al. (2024)	Single‐blind, parallel‐group RCT	32 randomized; 30 completed and analyzed; LIRT‐BFR, *n* = 15; HIRT, *n* = 15	First‐ever unilateral ischemic stroke	> 1 month after stroke. Mean time since stroke was 25.60 ± 29.89 months in LIRT‐BFR and 23.06 ± 31.45 months in HIRT. Subacute/non‐chronic participants: 7/15 and 6/15; chronic participants: 8/15 and 9/15, respectively	Participants could walk 10 m independently, with or without an assistive device, and had FAC > 3. Six participants used a walking aid. MAS < 1. Baseline FAC was 4.00 ± 0.84 in LIRT‐BFR and 3.93 ± 0.70 in HIRT. Baseline FMA‐LE and MMT values were not reported. Severity was not explicitly classified	Low‐intensity resistance training at 40% 1‐RM with BFR at 150–160 mmHg, combined with 20 min of aerobic training	High‐intensity resistance training at 80% 1‐RM, combined with 20 min of aerobic training	5 weeks; resistance training 3 times/week	LIRT‐BFR versus HIRT: 6‐MWT, 81.00 versus 62.91 m; MD = 18.09 m, *p* = 0.809. No significant differences were observed for 5TSTST, TUGT, Barthel Index, gait speed, or stride length.	Two HIRT participants reported elevated blood pressure, considered unrelated to the intervention. One LIRT‐BFR participant reported muscle pain that resolved within 12 h. No serious adverse event was reported
Feng et al. (2023)	Parallel‐group RCT	34 randomized; 29 completed and analyzed; BFR‐ET, *n* = 15; ET, *n* = 14	Ischemic and hemorrhagic stroke with unilateral hemiplegia	BFR‐ET: 12.27 ± 11.55 months; ET: 14.36 ± 15.45 months. The authors did not explicitly define the sample as acute, subacute, or chronic	Lower‐limb Brunnstrom Stages II–V. Participants had basic walking ability and required supervision during walking. In the BFR‐ET group, baseline MMT was reported as follows: hip flexion, 3 (3–3); hip extension, 1 (1–2); hip abduction, 2 (2–3); knee extension, 3 (2–4); knee flexion, 3 (2–3); ankle dorsiflexion, 2 (1–3); plantar flexion, 3 (2–3). Baseline FMA‐LE was 20 (14–23) in BFR‐ET and 24 (20.25–27.75) in ET. Baseline MBI was 69 (62–84) and 75 (65–91.25), respectively. Baseline TUGT was 41.52 (28.66–55.32) s and 28.96 (25.50–38.43) s, respectively. Severity was not explicitly classified	Exercise training combined with BFR‐ET at 160–200 mmHg; BFR‐ET was performed for 20 min once daily in addition to exercise training	Exercise training alone, 30 min twice daily	20 days	Both groups improved in FMA‐LE, TUGT, and MBI. BFR‐ET produced greater improvements in ankle plantar‐flexion active range of motion and hip‐abduction strength. Between‐group differences in overall FMA‐LE, TUGT, and MBI outcomes were not consistently significant	No thrombosis, syncope, or other adverse events were reported
Li et al. (2025)	RCT	34 participants; 17 per group	Ischemic and hemorrhagic stroke; control group: cerebral infarction, *n* = 6, and cerebral hemorrhage, *n* = 11; BFR group: cerebral infarction, *n* = 8, and cerebral hemorrhage, *n* = 9	Disease duration was 41.53 ± 33.50 days in the control group and 47.06 ± 42.26 days in the BFR group. The inclusion criterion was onset within 6 months. The authors did not explicitly label the participants as acute or subacute	Seated balance Level 2 was required. Participants were able to participate in cycle‐ergometer training. The article did not report baseline FMA‐LE, MMT, TUG, walking distance, or walking‐assistance requirements. Severity was not explicitly classified	Cycle‐ergometer training with individualized BFR at 40%–80% AOP; each session lasted 10 min, twice daily, 5 days/week	Cycle‐ergometer training with cuff pressure at 10% AOP	3 weeks	Compared with the control group, BFR improved rectus femoris thickness, rectus femoris cross‐sectional area, gastrocnemius pennation angle, 30‐s sit‐to‐stand performance, stretch‐test performance, and FMA‐LE	The authors reported no serious adverse events. Minor cuff discomfort or transient muscle soreness resolved without intervention
Bai et al. (2025)	Prospective, double‐blind, three‐arm RCT	66 enrolled; 60 completed and analyzed; BFR, LL, and HL groups, *n* = 20 each in the analyzed sample	First‐ever subcortical ischemic stroke with unilateral supratentorial lesion and hemiparesis	Stroke onset occurred within 2 weeks to 6 months before enrollment. Time post‐stroke was reported as median (IQR): BFR, 23.0 (27.0); LL, 29.0 (46.0); HL, 27.5 (54.0). The inclusion window indicates an early post‐stroke to subacute population, although the authors did not explicitly define the recovery stage.	Major flexor and extensor muscle groups of the affected lower limb had MMT ≥ 3. Participants had to cooperate with the fNIRS task. Baseline MMSE was 23.8 ± 4.2 in BFR, 24.2 ± 4.0 in LL, and 25.7 ± 4.0 in HL. Baseline FMA‐LE was not presented in Table 1. Patients with severe hemiplegia and muscle strength < 3 were excluded. The authors described the cohort as mild‐to‐moderate in the Discussion, but did not provide a separate severity distribution	Cycling at 30% 1‐RM with intermittent BFR at 200 mmHg	Matched low‐load cycling at 30% 1‐RM without BFR; high‐load cycling at 80% 1‐RM	4 weeks; 5 days/week	BFRT versus low‐load training: knee‐extensor peak torque, mean change 12.63 versus 1.45 N·m; MD = 11.18 N·m, *p* < 0.001. No significant difference was observed between BFRT and high‐load training. The high‐load group had a greater FMA‐LE improvement than the low‐load group (MD = 5.20, *p* = 0.023).	Six participants did not complete the study: one because of fNIRS motion artifacts, two in the HL group because of unbearable muscle soreness or weakness, and three because of dissociation during follow‐up. No additional BFR‐specific serious adverse event was reported
Qin et al. (2026)	Parallel‐group, assessor‐blinded RCT	82 recruited; 80 completed and analyzed; RR, *n* = 40; RR + BFRT, *n* = 40	Ischemic and hemorrhagic stroke. The abstract describes first‐ever stroke; the detailed inclusion criteria also allowed recurrent stroke without significant residual impairment	Disease duration was 45.7 ± 14.6 days in RR and 45.9 ± 13.9 days in RR + BFRT. This represents an early post‐stroke or subacute sample, although the authors did not explicitly define the recovery stage	Baseline FMA‐LE was 8.25 ± 1.82 in RR and 8.70 ± 2.70 in RR + BFRT. Baseline BBS was 27.85 ± 3.42 and 26.08 ± 5.12; MBI was 28.08 ± 5.95 and 29.73 ± 4.60; and 6MWT was 12.23 ± 4.40 m and 12.53 ± 4.19 m, respectively. Baseline MMT in RR and RR + BFRT, respectively, was: iliopsoas, 2.45 ± 0.50 and 2.33 ± 0.62; quadriceps, 2.33 ± 0.47 and 2.18 ± 0.55; hamstrings, 2.25 ± 0.59 and 2.10 ± 0.59; tibialis anterior, 1.05 ± 0.50 and 1.03 ± 0.86. MMSE was 21.88 ± 3.70 and 20.90 ± 4.10. Participants were conscious, cooperative, medically stable, and able to complete training; walking‐assistance requirements were not separately reported. Severity was not explicitly classified	Routine rehabilitation plus active low‐load BFRT at 160–170 mmHg and approximately 20%–30% estimated 1‐RM	Routine rehabilitation alone	8 weeks; 5 days/week	RR + BFRT versus RR: post‐intervention MDs were 2.07 points for FMA‐LE (*p* = 0.026), 3.18 points for BBS (*p* = 0.037), 8.05 points for MBI (*p* = 0.002), and 5.62 m for 6‐MWT (*p* = 0.001). Significant differences were also found for iliopsoas, quadriceps, and hamstring strength, but not tibialis anterior strength.	No serious adverse event occurred. One RR participant died from a non‐treatment‐related complication. One RR + BFRT participant was lost to follow‐up. No severe numbness, intervention‐requiring blood‐pressure fluctuation, excessive delayed‐onset muscle soreness, or symptoms suggestive of DVT were reported
Sun et al. (2024)	Three‐arm RCT	90 initially enrolled; 80 completed and analyzed: conventional rehabilitation, *n* = 25; WBV, n = 28; WBV + iBFR, *n* = 27	First‐ever ischemic and hemorrhagic stroke with hemiplegia; 56 cerebral infarctions and 24 cerebral hemorrhages	Patients were described as being in the convalescent stage and had disease duration within 6 months. Mean disease duration was 42.63 ± 21.48 days in WBV + iBFR, 51.75 ± 26.56 days in WBV, and 46.16 ± 22.97 days in the control group	Participants were 60–85 years old, had MMSE > 21, standing balance grade 2–3, and could walk independently or with a cane for at least 30 m. Baseline BBS was 39.70 ± 2.25 in the control group, 38.82 ± 2.28 in WBV, and 39.24 ± 2.24 in WBV + iBFR. Baseline 6MWT was 90.20 ± 31.60 m, 85.14 ± 21.32 m, and 86.07 ± 26.62 m, respectively. Baseline CB&M was 21.68 ± 13.18, 22.93 ± 10.08, and 20.07 ± 12.52, respectively. Baseline FMA‐LE, MMT, and Brunnstrom stage were not reported. The authors did not explicitly classify impairment severity	Conventional rehabilitation plus WBV and intermittent BFR at 140–200 mmHg; 3 min restriction followed by 1 min release	Conventional rehabilitation alone or conventional rehabilitation plus WBV	6 weeks; CB&M follow‐up at 1 and 3 months after discharge	After 6 weeks, both WBV and WBV + iBFR produced greater improvements in BBS, 6MWT distance, and CB&M scores than conventional rehabilitation, whereas no significant difference was observed between WBV + iBFR and WBV for these outcomes. At 1‐month follow‐up, both WBV and WBV + iBFR remained superior to conventional rehabilitation in CB&M scores, with no significant difference between the two active‐treatment groups. At 3‐month follow‐up, WBV + iBFR produced higher CB&M scores than WBV and both active‐treatment groups remained superior to conventional rehabilitation.	The article did not provide a specific adverse‐event count or a dedicated safety analysis. Therefore, safety should be recorded as not specifically reported
Kjeldsen et al. (2022)	Preliminary randomized crossover study	14 participants	Stroke; pathological type not specified in the study report	Chronic stroke population; the exact time since stroke was not consistently reported in the available text	Participants were described as mildly impaired and able to perform active ankle dorsiflexion. The study investigated tibialis‐anterior exercise. The operational definition of “mild impairment” and standardized baseline FMA‐LE, MMT, walking, or balance scores were not reported	Tibialis‐anterior exercise with BFR at approximately 80% systolic blood pressure, approximately 150 mmHg	The same exercise without BFR	Single‐session crossover experiment	No significant differences between BFR and non‐BFR conditions were found for EMG, TMS, SICI, ICF, or M‐max outcomes	The intervention was reported to be safe and well tolerated in this small sample
Feng et al. (2025)	Assessor‐blinded, block‐randomized parallel‐group RCT	40 randomized; BFRET, *n* = 20; ET, *n* = 20. Post‐treatment analysis included 20 BFRET and 18 ET participants	Ischemic and hemorrhagic stroke with unilateral hemispheric involvement	BFRET: median 7.0 months, IQR 4.0–23.5; ET: median 6.0 months, IQR 3.5–24.0. The sample was predominantly chronic according to the reported disease duration	Lower‐limb function above Brunnstrom stage II was required. Baseline FMA‐LE was 19.80 ± 5.99 in BFRET and 22.71 ± 6.84 in ET. Baseline total MMT was 16.10 ± 4.66 and 19.12 ± 6.08, respectively. Baseline TUGT was 39.96 ± 19.50 s and 34.77 ± 22.37 s. Participants could perform active limb exercise, transfer, standing‐balance, walking, and stair training according to ability. Exact walking‐assistance requirements were not reported. Baseline MMSE was reported as median 28 (IQR 26–29) and 25 (IQR 19–29), respectively. Severity was not explicitly classified	Exercise training plus BFR at 160–200 mmHg; 30 min/day	Exercise training alone; 30 min twice/day	4 weeks; 20 sessions	Both groups improved over time in total MMT, FMA‐LE, and TUGT. BFRET produced a greater improvement in total lower‐limb MMT than ET (between‐group *p* = 0.005), whereas no significant between‐group differences were found for FMA‐LE or TUGT. The article reported significant time‐by‐group interactions for hip‐flexion and plantar‐flexion MMT, although the abstract described hip‐flexion and plantar‐flexion outcomes.	No serious adverse events were reported. Safety monitoring included blood pressure, oxygen saturation, cuff pressure, limb color, limb dimension, fatigue, and numbness

Abbreviations: AOP, arterial occlusion pressure; BFRT, blood flow restriction training; BI, Barthel Index; BP, blood pressure; CB&M, Community Balance and Mobility Scale; EMG, electromyography; ET, exercise training; FMA‐LE, Fugl–Meyer Assessment of the Lower Extremity; HIRT, high‐intensity resistance training; HL, high‐load or high‐intensity training; ICF, intracortical facilitation; LIRT‐BFR, low‐intensity resistance training with blood flow restriction; LL, low‐load training; M‐max, maximum M‐wave; MBI, Modified Barthel Index; MMT, manual muscle testing; SICI, short‐interval intracortical inhibition; TMS, transcranial magnetic stimulation; TUGT, Timed Up and Go Test; WBV, whole‐body vibration.

Baseline motor impairment was also characterized inconsistently. Eligibility criteria generally required participants to retain sufficient postural control, lower‐limb strength, or voluntary motor function to complete active cycling, resistance, or gait‐related exercise (Bai et al. 2025; Feng et al. 2023; Feng et al. 2025; Kjeldsen et al. 2022; Li et al. 2025; Qin et al. 2026). Functional ambulation was explicitly required in some studies: participants had to be able to walk at least 10 m independently, with or without an assistive device, or at least 30 m independently or with a cane (Ahmed et al. 2024; Sun et al. 2024). Other studies enrolled participants across Brunnstrom Stages II–V or required lower‐limb muscle strength of at least grade 3, indicating variation in baseline motor recovery while excluding some patients with profound impairment (Bai et al. 2025; Feng et al. 2023; Feng et al. 2025). Nevertheless, standardized severity classifications and detailed baseline motor scores were not reported consistently, limiting direct comparisons of impairment severity across studies.

Collectively, the available evidence is most directly applicable to medically stable stroke survivors who retain sufficient voluntary lower‐limb motor control and functional capacity to participate actively in exercise (Ahmed et al. 2024; Bai et al. 2025; Feng et al. 2023; Feng et al. 2025; Kjeldsen et al. 2022; Li et al. 2025; Qin et al. 2026; Sun et al. 2024). Individuals with severe paresis, absent voluntary lower‐limb movement, nonambulatory status, or substantial mobility dependence were excluded or underrepresented, as reflected by eligibility requirements related to active movement, lower‐limb strength, sitting or standing balance, and walking capacity (Ahmed et al. 2024; Bai et al. 2025; Kjeldsen et al. 2022; Li et al. 2025; Sun et al. 2024). Therefore, the efficacy and safety of BFRT in patients with more severe motor and mobility limitations remain uncertain.

### 3.2 Risk of Bias of the Included Studies

The risk‐of‐bias assessments of the included RCTs are summarized in Figure 2. The assessment was conducted across seven domains, including random sequence generation, allocation concealment, blinding of participants and personnel, blinding of outcome assessment, incomplete outcome data, selective reporting, and other potential sources of bias. The risk‐of‐bias judgments varied across the included trials. Some domains were judged as low risk, whereas unclear or high risk was observed in domains for which the reporting of allocation procedures, blinding, or other methodological details was insufficient. Because blinding of participants and therapists is difficult in exercise‐based rehabilitation trials, this domain should be interpreted in the context of the intervention characteristics. Overall, the risk‐of‐bias assessment indicates that the findings should be interpreted cautiously, particularly when comparing the effects of BFRT with those of control interventions.

**FIGURE 2:**
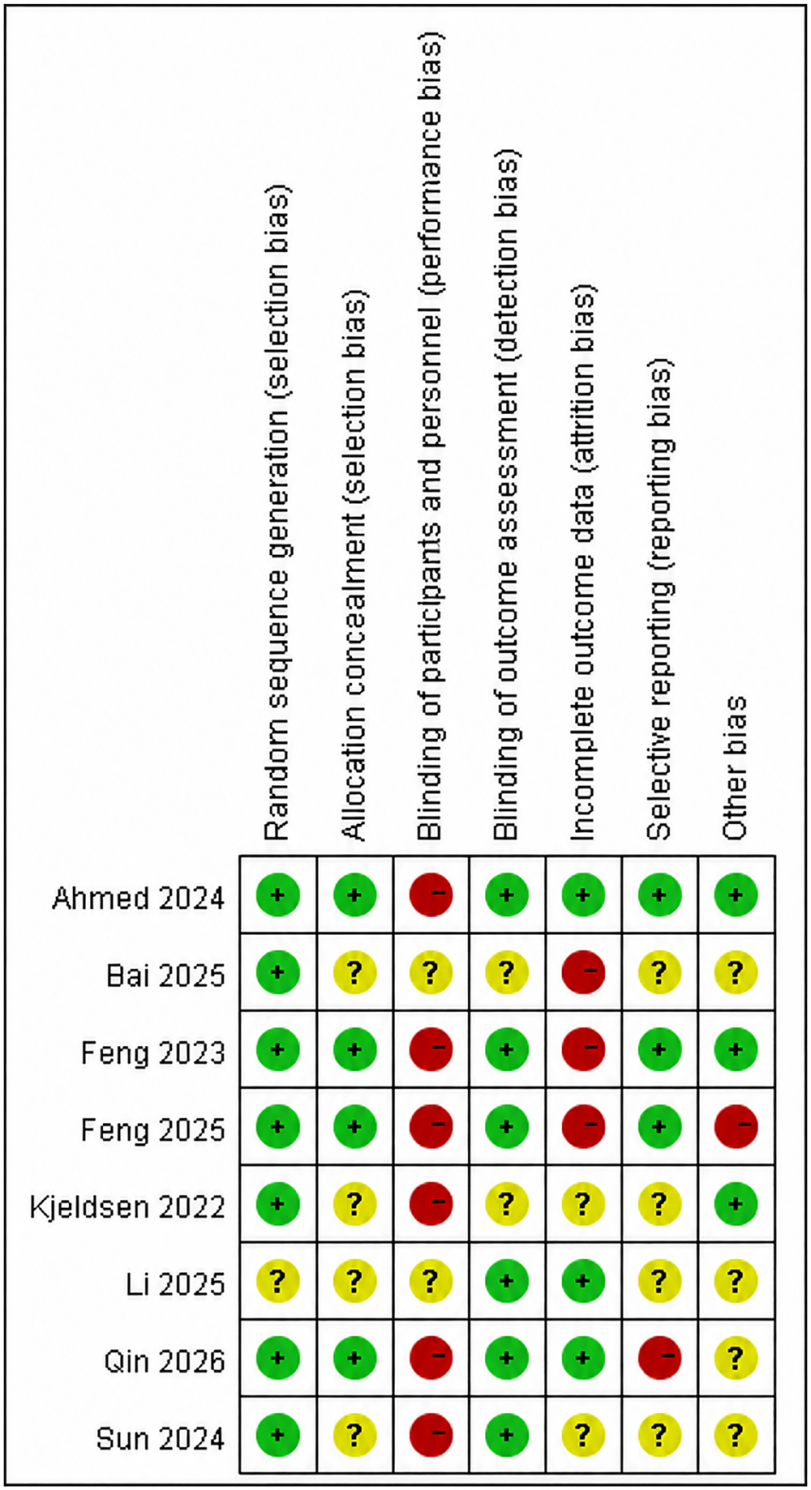
**Risk‐of‐bias assessment of the included randomized controlled trials using the Cochrane risk‐of‐bias tool**. The assessment included random sequence generation, allocation concealment, blinding of participants and personnel, blinding of outcome assessment, incomplete outcome data, selective reporting, and other bias. Green “+” indicates low risk of bias, red “−” indicates high risk of bias, and yellow “?” indicates unclear risk of bias.

### 3.3 Lower Extremity Function and Muscle Strength

Lower‐extremity motor and gait rehabilitation is a major component of post‐stroke care because motor impairment can restrict walking, independence, and participation in daily life (Alt Murphy et al. 2025). RCTs have evaluated BFRT as an approach to improving lower‐limb strength and motor function after stroke, although between‐group benefits have not been consistent across all outcomes (Ahmed et al. 2024; Bai et al. 2025; Feng et al. 2025; Qin et al. 2026).

An RCT (Ahmed et al. 2024) compared low‐intensity resistance training with blood flow restriction (LIRT‐BFR) to high‐intensity resistance training (HIRT) in 32 ischemic stroke survivors over a five‐week period. Both groups showed significant improvements in the 6‐minute walk test (6‐MWT), five‐times sit‐to‐stand test (5TSTST), timed up and go test (TUGT), and Barthel Index. In the study by Ahmed et al. (2024), the mean improvement in 6‐MWT distance was 81.00 m in the LIRT‐BFR group and 62.91 m in the HIRT group, corresponding to an unadjusted between‐group difference of 18.09 m (*p* = 0.809). Similarly, the between‐group differences were −0.46 s for the 5TSTST (*p* = 0.870), −2.76 s for the TUG test (*p* = 0.987), 0.33 for the Barthel Index (*p* = 0.881), 0.07 m/s for gait speed (*p* = 0.212), and 5.18 cm for stride length (*p* = 0.801). None of these between‐group comparisons reached statistical significance. No serious adverse events were reported.

In an RCT comparing exercise training combined with blood flow restriction (BFRET) with exercise training alone (ET), the change in total manual muscle testing (MMT) score differed significantly between groups (*p* = 0.005). Significant time‐by‐group interactions were also observed for hip‐flexion and plantar‐flexion strength (both *p* = 0.04), as well as for *Vastus lateralis* and tibialis anterior muscle thickness (*p* = 0.03 and *p* = 0.04, respectively). In contrast, FMA‐LE scores improved over time in both groups, but the time‐by‐group interaction was not statistically significant (*p* = 0.13). TUGT performance also improved over time without a significant time‐by‐group interaction (*p* = 0.38). Because numerical between‐group MDs were not reported for all outcomes, these findings should be interpreted cautiously (Feng et al. 2025).

A large assessor‐blinded RCT enrolled 80 stroke patients and compared eight weeks of routine rehabilitation (RR) plus BFRT with RR alone. At post‐intervention assessment, the RR + BFRT group had higher scores than the RR group for FMA‐LE (16.60 ± 4.61 vs. 14.53 ± 3.47; MD = 2.07, *p* = 0.026), BBS (44.08 ± 8.01 vs. 40.90 ± 5.07; MD = 3.18, *p* = 0.037), MBI (61.18 ± 11.46 vs. 53.13 ± 11.31; MD = 8.05, *p* = 0.002), and 6‐MWT distance (37.85 ± 8.72 vs. 32.23 ± 6.14 m; MD = 5.62 m, *p* = 0.001). Significant between‐group differences were also observed for iliopsoas strength (MD = 0.35, *p* = 0.022), quadriceps strength (MD = 0.35, *p* = 0.027), and hamstring strength (MD = 0.40, *p* = 0.013), but not for tibialis anterior strength (MD = 0.05, *p* = 0.774). These findings are based on a single trial and require confirmation in larger studies (Qin et al. 2026).

A three‐arm RCT randomized 60 ischemic stroke patients to BFRT (30% 1‐RM with intermittent 200‐mmHg occlusion), matched low‐load training (30% 1‐RM), or high‐load training (80% 1‐RM) for four weeks. Compared with low‐load training, BFRT produced a greater improvement in knee‐extensor peak torque, with mean changes of 12.63 N·m and 1.45 N·m, respectively, corresponding to an MD of 11.18 N·m (*p* < 0.001). No statistically significant difference was observed between BFRT and high‐load training for knee‐extensor peak torque (*p* > 0.05). For FMA‐LE, the mean changes were 7.25 points in the BFRT group, 2.70 points in the low‐load group, and 7.90 points in the high‐load group. The high‐load group showed a greater improvement than the low‐load group (MD = 5.20, *p* = 0.023), whereas the difference between BFRT and high‐load training was not statistically significant. No significant between‐group difference was observed for rectus femoris cross‐sectional area (*p* > 0.05) (Bai et al. 2025). A systematic review (Jønsson et al. 2024) evaluated the safety and efficacy of BFR exercise in individuals with neurological disorders, including stroke, and found that BFRT appears safe and well‐tolerated, though methodological biases and heterogeneity limit definitive conclusions. As summarized in Table 1, the clinical studies included in this review primarily focused on lower‐limb strength, gait, balance, and functional mobility; none directly assessed upper‐limb motor recovery or upper‐limb spasticity after stroke.

### 3.4 Balance and Gait

Improvements in muscle strength may support better balance and gait performance; however, gains in strength do not necessarily translate directly into faster walking speeds or improved functional mobility, because these outcomes also depend on task‐specific practice, motor control, coordination, sensory function, and walking endurance. Several trials reported statistically significant improvements in walking capacity, functional mobility, or balance after interventions that included BFRT. However, the presence and magnitude of between‐group differences varied across studies, and statistical significance alone does not establish clinical importance. Gait speed is associated with functional walking status and has been used to distinguish household, limited community, and community ambulation after stroke (Perry et al. 1995). A mean within‐group increase in gait speed of 0.19 m/s was observed after low‐intensity resistance training combined with blood‐flow restriction. However, the corresponding improvement was 0.12 m/s with HIRT, resulting in a between‐group MD of 0.07 m/s that was not statistically significant (*p* = 0.212) (Ahmed et al. 2024). Although this change exceeded the 0.16 m/s minimal clinically important difference previously estimated in ambulatory individuals with subacute stroke and severe gait impairment (Tilson et al. 2010), the applicability of this threshold to the population studied by Ahmed et al. remains uncertain. Another study reported statistically significant within‐group improvements in walking speed and stride length after BFRT (Li et al. 2025); however, the evidence for a blood flow restriction‐specific between‐group benefit should be interpreted according to the reported comparative analyses. A statistically significant post‐intervention difference in Berg Balance Scale scores was observed between routine rehabilitation combined with BFRT and routine rehabilitation alone (44.08 ± 8.01 vs. 40.90 ± 5.07; MD = 3.18, *p* = 0.037), providing preliminary evidence of an additional benefit for balance performance. However, the physiological and neural mechanisms underlying this finding were not directly assessed, and the result, which was derived from a single trial, requires confirmation in larger, adequately powered studies (Qin et al. 2026).

### 3.5 Cognitive and Psychological Outcomes

Post‐stroke depression affects approximately one‐third of stroke survivors and is associated with poorer functional outcomes and quality of life (Towfighi et al. 2017). In the available clinical evidence, anxiety and depressive symptoms were assessed in one RCT. Improvements in these outcomes were observed within both the LIRT‐BFR group and the HIRT group; however, no significant between‐group differences were found (Ahmed et al. 2024). These findings suggest that BFRT may be feasible for patients with psychological symptoms, but they do not establish a specific advantage over HIRT. Moreover, cognitive outcomes have not been directly evaluated in the available clinical BFRT trials involving stroke survivors. Therefore, the potential effects of BFRT on post‐stroke cognitive function remain uncertain.

Preclinical evidence suggests that BFRT may have neuroprotective effects. The role of ferroptosis in cardiovascular disease and ischemic stroke has increasingly been recognized, with lipid peroxidation contributing to neuronal damage (Castillo‐González and González‐Rey 2025; Haupt et al. 2023). However, evidence linking BFRT to cognitive protection through mechanisms such as SIRT1 activation and suppression of ferroptosis is currently derived from preclinical studies and has not been validated in human stroke survivors (Zhou et al. 2025).

## 4 Mechanistic Underpinnings of BFRT in Stroke Recovery

Understanding the multiscale mechanisms of BFRT informs protocol design and patient stratification. It is important to note that much of the mechanistic understanding of BFRT in stroke recovery is derived from preclinical models or healthy populations. Direct evidence in stroke survivors remains sparse, and causal inferences from current human studies are limited by study design, sample size, and lack of mechanistic validation. Accordingly, the following mechanisms should be interpreted as hypotheses that warrant further investigation, rather than established pathways in post‐stroke BFRT.

### 4.1 Local Muscular Adaptations

BFRT‐induced hypertrophy primarily arises from acute intramuscular metabolic stress. Partial vascular occlusion restricts venous return, fostering a hypoxic, acidic environment that triggers the accumulation of lactate, hydrogen ions, and inorganic phosphate (Huang et al. 2025a). This metabolic milieu may promote motor‐unit recruitment, including the recruitment of higher‐threshold motor units, and anabolic signaling even at relatively low external loads; however, these mechanisms have not been directly confirmed in stroke survivors (Pearson and Hussain 2015; Patterson et al. 2019). These proposed mechanisms have been described primarily in healthy or non‐stroke populations, and their relevance to stroke survivors remains uncertain (Slysz et al. 2016; Lixandrão et al. 2018; Centner et al. 2019; Lorenz et al. 2021). Metabolite buildup may also stimulate the systemic release of growth hormone and insulin‐like growth factor‐1 (IGF‐1), while occlusion‐related cellular swelling may activate mammalian target of rapamycin (mTOR) signaling independently of high mechanical tension (Huang et al. 2025a). Empirical support includes ultrasonographic data showing increased muscle cross‐sectional area in stroke patients (Li et al. 2025).

### 4.2 Hemodynamic and Vascular Responses

BFRT can elicit acute cardiovascular responses. Canine models indicate that low‐intensity BFRT triggers the muscle metaboreflex, sharply increasing heart rate, stroke volume, cardiac output, and mean arterial pressure (Mannozzi et al. 2023). Human evidence indicates that acute cardiovascular responses to blood‐flow‐restricted resistance exercise depend on the comparison condition and protocol, with low‐load BFRT not necessarily producing greater cardiac responses than high‐load exercise (Suggitt et al. 2024; Huang et al. 2025a). This hypertensive response, mediated by compression and the metaboreflex, potentially enhances perfusion, while histamine receptors facilitate post‐exercise hypotension (Seo et al. 2024). Cardiovascular responses to BFRT depend on the cuff pressure, cuff width, exercise modality, exercise intensity, occlusion duration, and characteristics of the participant. Accordingly, hemodynamic responses reported in healthy or older populations cannot be directly extrapolated to stroke survivors (Zhang et al. 2022b; Huang et al. 2025a; Miller et al. 2021).

After stroke, autonomic cardiovascular control may be disrupted, including altered sympathovagal balance, reduced baroreflex sensitivity, and impaired regulation of heart rate and blood pressure during exercise. However, the muscle metaboreflex has not been systematically characterized in stroke survivors undergoing BFRT, and its alteration is unlikely to be uniform across patients. A blunted metaboreflex could produce an inadequate compensatory increase in sympathetic activity, cardiac output, and arterial pressure during restricted exercise, potentially contributing to reduced exercise tolerance, insufficient perfusion, dizziness, or hypotension after cuff release. Conversely, an overactive metaboreflex could amplify BFRT‐induced sympathetic vasoconstriction and pressor responses, thereby increasing cardiac workload and the risk associated with excessive blood‐pressure elevation. These consequences remain physiologically plausible rather than directly demonstrated in post‐stroke BFRT trials. Therefore, patients with suspected autonomic dysfunction or exaggerated exercise blood‐pressure responses require individualized cuff‐pressure prescription and close monitoring of blood pressure, heart rate, symptoms, and post‐release recovery.

Of particular relevance to stroke are cortical hemodynamic responses during exercise. In a randomized fNIRS study, cortical Oxyhemoglobin (HbO) concentrations were assessed during a cycling task in stroke patients assigned to BFRT at 30% 1‐RM, matched low‐load training at 30% 1‐RM, or high‐load training at 80% 1‐RM. The fNIRS assessment used a block design consisting of 30 s of rest, five 20‐s pedaling cycles, and a final 30‐s rest period, and the fNIRS data were collected during the cycling sessions. During the first session, HbO concentrations were significantly greater in the blood flow restriction and high‐load groups than in the matched low‐load group (*p* = 0.003 and *p* = 0.020, respectively), with no significant difference between the blood flow restriction and high‐load groups (*p* = 0.511). After the four‐week intervention, the pre‐to‐post change in HbO concentration was also greater in the blood flow restriction and high‐load groups than in the matched low‐load group (*p* = 0.010 and *p* = 0.028, respectively), whereas no significant difference was found between the blood flow restriction and high‐load groups (*p* = 0.711). Changes in HbO in the primary motor cortex and premotor cortex–supplementary motor area of the affected hemisphere were positively correlated with changes in knee‐extensor peak torque and FMA‐LE scores. Therefore, the reported HbO findings represent cortical hemodynamic responses assessed during the cycling task rather than resting‐state HbO measurements. Because fNIRS provides an indirect measure of cortical hemodynamic activity, these findings should not be interpreted as direct evidence of increased cerebral blood flow or cortical reorganization (Bai et al. 2025).

The mechanisms underlying the observed cortical HbO responses remain uncertain. Remote ischemic conditioning (RIC) involves repeated brief cycles of ischemia and reperfusion applied to a limb and is conceptually distinct from BFRT, which combines partial vascular restriction with active exercise. Evidence from RIC should therefore not be interpreted as direct evidence that BFRT alters cerebral blood flow or cerebral autoregulation. Cerebral blood flow regulation is closely related to blood–brain barrier integrity and neural homeostasis (Haupt et al. 2023; Sun et al. 2023; Wang et al. 2025). In experimental ischemic stroke, endothelial sphingosine‐1‐phosphate receptor 1 (S1P1) signaling has been shown to counteract infarct expansion, highlighting the importance of endothelial signaling in the regulation of post‐ischemic vascular responses (Nitzsche et al. 2021). Whether BFRT influences S1P1 signaling or related endothelial pathways in stroke survivors remains unknown. Nevertheless, whether BFRT affects cerebral autoregulation, collateral cerebral perfusion, or blood–brain barrier integrity in stroke survivors has not been directly established. Further studies incorporating direct measurements of cerebral blood flow and cerebrovascular reactivity are required to clarify these potential effects.

### 4.3 Cellular Signaling and Ferroptosis

Ferroptosis is an iron‐dependent form of regulated cell death characterized by the accumulation of lipid peroxides and the failure of cellular antioxidant defenses, particularly the glutathione‐dependent GPX4 system. Iron‐dependent lipid peroxidation and oxidative stress have been implicated in cardiovascular and ischemic tissue injury, providing a biological context for the potential involvement of ferroptosis after ischemic stroke (Chen et al. 2021; Zhang et al. 2022a). After ischemic stroke, iron dysregulation, mitochondrial dysfunction, and oxidative stress associated with ischemia–reperfusion may promote lipid peroxidation and ferroptotic damage in neural cells. This process is important because it can contribute to secondary injury beyond the initial ischemic insult, potentially aggravating neuronal and synaptic damage and impairing functional recovery. Preclinical evidence suggests that BFRT may attenuate stroke‐associated ferroptotic signaling through upregulation of the SIRT1 pathway, with accompanying reductions in hippocampal synaptic damage and cognitive deficits in MCAO rats (Zhou et al. 2025). However, these findings are derived from an animal model, and whether BFRT modulates ferroptosis in human stroke survivors remains unknown. Thus, ferroptosis represents a plausible cellular mechanism linking BFRT to neural protection, but this interpretation requires validation in clinical studies.

### 4.4 Extracellular Vesicles and Systemic Messengers

Extracellular vesicles (EVs) increasingly serve as intercellular communication mediators. Circulating microRNA profiles have been associated with stroke‐related biological processes and clinical characteristics, although the specific functions of these microRNAs remain incompletely understood (Fullerton et al. 2022). Experimental evidence from remote ischemic conditioning and exercise models suggests that plasma EVs may contribute to endothelial protection and exercise‐induced ischemic tolerance; however, whether BFRT induces comparable EV‐mediated responses remains unknown. These findings raise the possibility that BFRT may stimulate the release of extracellular vesicles carrying potentially protective molecular cargo, although whether these vesicles selectively target ischemic brain tissue remains to be determined (Gu et al. 2022). Although the specific cargo of BFR‐induced EVs remains to be fully elucidated, their potential therapeutic relevance in stroke represents a promising avenue for future research. Exosomal microRNA delivery has also been investigated as a potential strategy for modulating neuroinflammation after subarachnoid hemorrhage; however, this evidence does not directly establish a similar mechanism in BFRT‐treated ischemic stroke survivors (Sheng et al. 2025).

### 4.5 Neurovascular Unit and Blood‐Brain Barrier

The neurovascular unit (NVU), which integrates endothelial, pericytic, astrocytic, and neuronal components, plays a critical role in maintaining cerebral homeostasis and modulating ischemic injury (Sun et al. 2023). Pericytes, for instance, have been implicated in regulating reperfusion dynamics after cerebral ischemia (Dalkara et al. 2025). However, the restrictive nature of the blood‐brain barrier (BBB) often limits therapeutic access to neural tissues (Haupt et al. 2023; Wang et al. 2025).

It is currently unknown whether BFRT directly affects BBB integrity in stroke patients. Indirect evidence from post‐thrombectomy imaging studies indicates that reperfusion‐related hemodynamic changes may be accompanied by blood–brain barrier disruption or contrast staining beyond the infarcted tissue (Abdalkader et al. 2024; Mutke et al. 2025). However, these findings do not establish that BFRT or remote ischemic conditioning produces comparable effects on blood–brain barrier integrity. Additionally, molecules such as Nogo‐A, which can be secreted in extracellular vesicles, have been shown to modulate vascular permeability in experimental models (Rust et al. 2024). Whether similar vesicle‐mediated mechanisms are engaged by BFRT, and whether they translate into clinically meaningful BBB protection after stroke, remains speculative and requires direct investigation.

The proposed local, systemic, and central mechanisms of BFRT in stroke rehabilitation are summarized in Figure 3.

**FIGURE 3:**
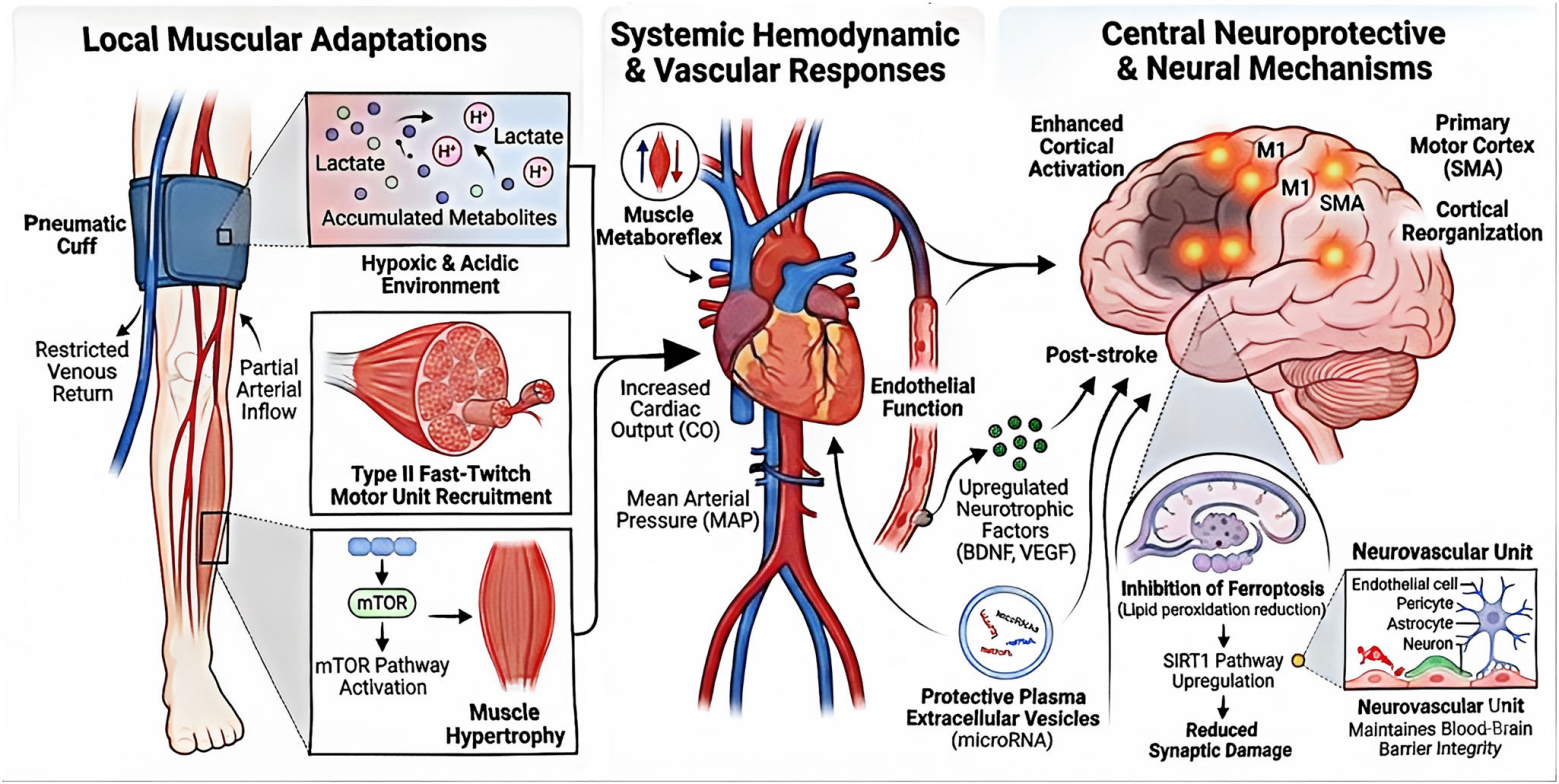
**Theoretical framework of the potential mechanisms of BFRT in stroke rehabilitation**.This figure illustrates the hypothesized local and systemic mechanisms through which BFRT may influence stroke rehabilitation. Locally, BFRT may create a hypoxic and metabolically stressful environment that promotes recruitment of fast‐twitch muscle fibers and activation of the mTOR pathway. Systemically, metabolite accumulation may induce vascular and neural responses that could influence neurovascular integrity and functional recovery. The pathways shown are hypothetical and hypothesis‐generating. They are based primarily on indirect evidence from non‐stroke populations, related conditioning paradigms, and preclinical models and have not been systematically validated in human stroke survivors. The proposed pathways should not be interpreted as established causal relationships. BBB: blood‐brain barrier; BDNF: brain‐berived neurotrophic factor; CO: cardiac output; M1: primary motor cortex; MAP: mean arterial pressure; miRNA: microRNA; mTOR: mammalian target of rapamycin; SIRT1: sirtuin 1; SMA: supplementary motor area; VEGF: vascular endothelial growth factor.

## 5 Safety Considerations in Stroke Populations

BFRT may cause transient increases in blood pressure, heart rate, sympathetic activity, and cardiac workload. Potential cardiovascular concerns include dizziness, presyncope, dyspnea, chest discomfort, arrhythmia, myocardial ischemia, and abnormal blood‐pressure recovery after cuff release. Vascular risks include venous stasis, deep‐vein thrombosis, thromboembolism, impaired limb perfusion, and local ischemia, particularly in patients with uncontrolled hypertension, cardiovascular disease, peripheral arterial disease, previous or active venous thrombosis, or significant cerebrovascular stenosis. Local complications may include pain, numbness, paresthesia, nerve compression, skin irritation, bruising, hematoma, and changes in limb color or temperature. Although low‐load BFRT does not consistently appear to cause clinically meaningful increases in muscle‐damage markers, excessive exercise intensity, cuff pressure, or occlusion duration may increase the risk of severe fatigue and muscle injury, particularly in medically vulnerable individuals (De Queiros et al. 2021, Patterson et al. 2019). Patients receiving anticoagulants or dual‐antiplatelet therapy may require additional assessment because of the potential for bruising or bleeding.

Given these potential risks, risk stratification before BFRT is particularly important for individuals with cardiovascular disease, peripheral vascular disease, thromboembolic risk, uncontrolled hypertension, impaired sensation, or complex medical histories (Cognetti et al. 2022; Nascimento et al. 2022; Patterson et al. 2019).

The available studies enrolled selected and medically stable stroke survivors, and their exclusion criteria varied. One study excluded participants with severe cognitive impairment (mini‐mental state examination score < 20), large cortical lesions involving more than one‐third of the middle cerebral artery territory, severe carotid stenosis (> 90%), severe cerebral arterial stenosis (> 75%), generalized medical instability, or severe scalp dermatitis (Bai et al. 2025). Another study excluded participants with significant cognitive impairment, unwillingness to participate, significant cardiovascular disease, blood pressure > 160/100 mmHg, peripheral arterial disease, lower‐limb venous thrombosis, complex peripheral neuropathy, anticoagulant or dual‐antiplatelet therapy, or other serious systemic diseases (Li et al. 2025).

The remaining studies required sufficient motor function, lower‐limb strength, postural control, cognition, communication, or exercise capacity to complete the intervention and assessments (Ahmed et al. 2024; Feng et al. 2023; Feng et al. 2025; Kjeldsen et al. 2022; Qin et al. 2026; Sun et al. 2024). However, detailed exclusion criteria relating to cardiovascular disease, thromboembolic risk, coagulation status, sensory impairment, autonomic dysfunction, and medication use were not consistently reported. Therefore, these criteria should be considered incompletely reported rather than assumed to be absent. The lack of serious reported adverse events indicates short‐term tolerability in selected participants but does not establish universal safety across all stroke survivors.

Before BFRT, screening should include medical stability, resting blood pressure, cardiovascular and thromboembolic history, peripheral vascular status, bleeding risk, medication use, sensory and cognitive function, communication ability, and exercise capacity (Billinger et al. 2014; Nascimento et al. 2022). Because pressure prescription methods and the degree of vascular restriction can substantially influence the physiological and perceptual responses to BFRT, the restriction procedure should be standardized and clearly reported (Aniceto and da Silva Leandro 2022; Cummings and Madhavan 2024; Jønsson et al. 2024). During training, clinicians should monitor blood pressure, heart rate, symptoms, limb color and temperature, pain, numbness, and recovery after cuff release. Training should be stopped if chest pain, marked dyspnea, presyncope or syncope, new neurological symptoms, severe limb pain, persistent numbness, abnormal limb discoloration, or an excessive hemodynamic response occurs. Future studies should report study‐specific exclusion criteria, adverse events, and treatment discontinuations in sufficient detail (Cummings and Madhavan 2024; Jønsson et al. 2024).

## 6 BFRT in the Context of Other Rehabilitation Modalities

Rather than substituting traditional protocols, BFRT may serve as an adjunct to conventional rehabilitation, although its additional clinical benefit remains uncertain. Benchmarking its utility against concurrent modalities clarifies the parameters for optimal clinical integration.

### 6.1 Comparison With High‐Load Resistance Training

HLRT can promote strength and hypertrophic adaptations, but some stroke survivors may be unable to tolerate high external loads because of paresis, impaired motor control, reduced exercise tolerance, or cardiovascular comorbidity. BFRT may provide a low‐load alternative or adjunct for selected patients; however, current evidence is insufficient to establish its equivalence or superiority to HLRT. Low‐load BFRT may induce adaptations through metabolic stress, motor‐unit recruitment, and anabolic signaling, but these mechanisms have not been directly confirmed in stroke survivors (Pearson and Hussain 2015; Patterson et al. 2019). Evidence from non‐stroke populations suggests that low‐load BFRT can stimulate strength and hypertrophic adaptations, but the effects are protocol‐dependent and should not be interpreted as evidence of equivalence with HLRT (Slysz et al. 2016; Lixandrão et al. 2018; Centner et al. 2019).

### 6.2 Comparison With Remote Ischemic Conditioning

RIC involves repeated brief cycles of ischemia and reperfusion in a limb, typically without concurrent exercise. Unlike RIC, BFRT combines vascular occlusion with active motor training. Although RIC and BFRT are distinct interventions, recent studies suggest that ischemic conditioning may have beneficial effects on motor function after stroke. Two weeks of ischemic conditioning improved walking speed and reduced neuromuscular fatigability in chronic stroke survivors (Durand et al. 2019), whereas a single session increased paretic‐leg strength through improved volitional muscle activation (Hyngstrom et al. 2018). However, the effects of BFRT on corticospinal transmission and other neurophysiological outcomes after stroke remain unclear. Further research should therefore directly examine corticospinal transmission, cortical excitability, motor‐unit recruitment, and functional recovery in stroke survivors receiving BFRT.

Recent reviews have described RIC as a potentially feasible adjunct in stroke recovery, but its mechanisms, optimal dosing, and clinical efficacy remain uncertain. Because RIC is performed without concurrent active exercise, findings from RIC studies should not be treated as direct evidence for BFRT (Keevil et al. 2024, Hess et al. 2022).

### 6.3 Integration With Task‐Specific Training

Rehabilitation aims to maximize functional independence. Evidence from post‐stroke physical therapy supports the importance of intensive, repetitive, and task‐oriented practice for improving motor function and walking ability, providing a rationale for considering BFRT as a possible adjunct rather than a replacement for functional rehabilitation (Veerbeek et al. 2014). Although BFRT has traditionally been confined to isolated strengthening, it may be integrated into task‐specific paradigms, such as treadmill gait or upper‐limb reaching. This approach would theoretically combine the metabolic stimulus of BFRT with the neuroplastic benefits of functional practice. However, to date, no study has directly validated this integrative strategy in stroke populations. Therefore, while the mechanistic rationale is plausible, the proposed combination remains a hypothesis that requires direct experimental testing. Although gamified upper‐limb rehabilitation has been investigated after stroke (Alberti et al. 2022), its integration with BFRT has not been evaluated. Future studies could examine whether task‐specific or gamified training can be safely combined with BFRT.

## 7 Discussion

Several small randomized trials have reported improvements in lower‐limb strength, gait, balance, or functional mobility following interventions that included BFRT. However, between‐group findings have been inconsistent, and the available studies do not establish that BFRT is superior or equivalent to high‐intensity training. This is clinically relevant because many stroke patients cannot tolerate high mechanical loads due to paresis or cardiovascular comorbidities. Proposed mechanisms of low‐load BFRT include local metabolic stress, increased motor‐unit recruitment, and activation of anabolic signaling pathways associated with muscle hypertrophy (Pearson and Hussain 2015, Patterson et al. 2019). However, these mechanisms have not been directly confirmed in stroke survivors. BFRT may activate the muscle metaboreflex and induce acute cardiovascular responses even during low‐intensity exercise (Mannozzi et al. 2023). However, whether this reflex is blunted or exaggerated after stroke has not been established in patients undergoing BFRT. Either alteration could affect hemodynamic tolerance: an inadequate response may compromise compensatory perfusion or blood‐pressure recovery, whereas an excessive response may amplify sympathetic vasoconstriction and exercise‐induced hypertension. In stroke survivors, cortical HbO responses in motor‐related regions were assessed during a cycling task using a rest–pedaling block‐design fNIRS paradigm. During the first cycling session, HbO concentrations were significantly greater in the blood flow restriction and high‐load groups than in the matched low‐load group. After four weeks, the pre‐to‐post changes in HbO were also greater in the blood flow restriction and high‐load groups than in the matched low‐load group. Thus, the reported HbO findings reflect cortical hemodynamic responses assessed during cycling rather than resting‐state HbO measurements. Because fNIRS measures cortical hemodynamic activity indirectly, these findings should not be interpreted as direct evidence of increased cerebral perfusion or BFRT‐induced cortical reorganization (Bai et al. 2025).

Several domains in the risk‐of‐bias assessment were judged as unclear or high risk, particularly when randomization, allocation concealment, blinding, or outcome‐reporting procedures were insufficiently described. These concerns reduce confidence in the estimated treatment effects and limit the strength of conclusions regarding the specific contribution of BFRT. The trials differed in stroke stage, baseline motor impairment, intervention protocols, cuff‐pressure procedures, comparator conditions, and outcome measures. Although blinding of participants and therapists is difficult in exercise‐based rehabilitation trials, insufficient reporting in these domains may reduce confidence in the estimated treatment effects. Several outcomes were supported by single trials or unadjusted between‐group comparisons, and numerical change scores were not consistently reported. Therefore, the observed improvements should be regarded as preliminary evidence rather than definitive proof of a specific BFRT effect.

However, the clinical applicability and generalizability of the current evidence are constrained by the characteristics of the populations studied. The available trials primarily enrolled medically stable stroke survivors who retained sufficient voluntary motor control, postural stability, lower‐limb strength, or walking capacity to participate actively in exercise (Ahmed et al. 2024; Bai et al. 2025; Feng et al. 2023; Feng et al. 2025; Kjeldsen et al. 2022; Li et al. 2025; Qin et al. 2026; Sun et al. 2024). Participants with severe paresis, absent voluntary lower‐limb movement, nonambulatory status, or substantial mobility dependence were excluded or underrepresented. Moreover, inconsistent reporting of time since stroke and baseline motor impairment prevents reliable determination of whether the effects of BFRT differ between recovery stages or according to impairment severity. This issue may be particularly important because post‐stroke recovery is associated with a time‐dependent window of vascular plasticity that is coupled to behavioral recovery (Williamson et al. 2020). Whether BFRT produces different effects when initiated during this window or at later stages of recovery remains unknown. Consequently, the current findings are most applicable to selected stroke survivors who can actively participate in exercise and should be generalized cautiously to patients with more severe motor or mobility limitations. The available safety findings should likewise be interpreted within this selected population and should not be generalized to all stroke survivors. Baseline blood flow in the paretic limb and autonomic function were not consistently assessed or reported in the included studies. Therefore, the safety of BFRT remains uncertain in individuals with markedly reduced resting blood flow in the paretic limb or autonomic dysfunction that may impair blood‐pressure recovery following exercise or cuff release. The certainty and generalizability of the current evidence remain limited because of small sample sizes, methodological limitations, protocol heterogeneity, and limited follow‐up (Jønsson et al. 2024). Future trials should recruit participants across a broader range of stroke stages and motor impairment severities and consistently report time since stroke, standardized baseline motor scores, ambulatory status, and functional eligibility criteria. Prespecified subgroup analyses are also needed to determine whether the efficacy and safety of BFRT differ according to recovery stage and baseline functional status. BFRT protocols varied substantially in cuff pressure, pressure individualization, occlusion pattern, training load, and exercise modality across the included studies, limiting direct comparisons and preventing the identification of an optimal protocol (Ahmed et al. 2024; Bai et al. 2025; Feng et al. 2025; Kjeldsen et al. 2022; Li et al. 2025; Qin et al. 2026; Sun et al. 2024). Intervention periods were generally short, ranging from a single experimental session to eight weeks, and follow‐up assessments were limited. Therefore, the long‐term durability of the reported functional improvements remains uncertain. Mechanistic evidence, such as that concerning ferroptosis, extracellular vesicles, and SIRT1, is derived almost exclusively from animal models and remains unvalidated in human stroke survivors. Moreover, none of the included studies directly assessed cognitive outcomes after BFRT. Although anxiety and depressive symptoms were assessed in one clinical study, the available evidence is insufficient to establish a specific antidepressant effect of BFRT over the comparator intervention (Ahmed et al. 2024). Accordingly, cognitive protection and antidepressant effects should not be considered established clinical benefits of BFRT at present. The available clinical evidence is also primarily limited to lower‐limb rehabilitation. None of the studies included in this review directly assessed upper‐limb motor recovery or upper‐limb spasticity after stroke. Therefore, the present findings should not be extrapolated to upper‐limb rehabilitation or spasticity management. This gap is consistent with previous reviews reporting limited and heterogeneous evidence for BFRT in neurological populations (Jønsson et al. 2024). Future RCTs should directly evaluate upper‐limb motor function and spasticity using standardized outcome measures and adequately reported BFRT protocols. Future trials should also include validated cognitive assessments and standardized measures of post‐stroke depression as prespecified secondary outcomes, with appropriate comparator groups to determine whether any observed changes exceed those attributable to general exercise or routine rehabilitation.

These limitations directly inform future research priorities. First, protocol standardization is essential. Future trials should prioritize individualized pressure prescription based on arterial or limb occlusion pressure (LOP) and report cuff width, cuff placement, exercise intensity, occlusion pattern, and applied pressure in sufficient detail (Patterson et al. 2019). Dose‐response studies are also needed to determine the optimal combination of occlusion pressure and exercise intensity. Comprehensive preparticipation screening and cardiovascular risk assessment should be incorporated into future trial protocols, particularly for medically complex stroke survivors (Nascimento et al. 2022). In patients with suspected vascular or autonomic impairment, future studies should also consider assessing limb perfusion, orthostatic symptoms, and blood‐pressure responses to exercise and cuff release. Second, long‐term RCTs (≥ 1 year follow‐up) are needed to assess durability of functional gains and long‐term safety, ideally including sham BFRT and active comparators, ideally including sham BFRT and active comparators. Third, mechanistic validation in humans should be embedded in such trials using multimodal neuroimaging and molecular assays (e.g., neurotrophic factors, microRNAs, and ferroptosis markers). Fourth, dedicated upper‐limb trials should include standardized measures of motor function, spasticity, and activities of daily living, while cognitive outcomes should be evaluated separately. Finally, technological innovations (wearable sensors, tele‐rehabilitation) offer a route to home‐based BFRT, but implementation and cost‐effectiveness studies are required before scaling.

In summary, BFRT is a mechanistically plausible low‐load intervention for stroke rehabilitation, but its evidence base remains too fragmented for routine clinical use. Advancing toward clinical integration requires LOP‐based standardization, long‐term placebo‐controlled trials, human mechanistic confirmation, and exploration of upper‐limb and cognitive outcomes. Until then, BFRT should be considered an experimental approach applied within rigorous research settings.

## 8 Conclusion

BFRT represents a promising low‐load intervention for post‐stroke rehabilitation. RCTs have reported improvements in lower‐limb strength, gait, and balance following BFRT‐based interventions; however, the evidence is insufficient to determine whether BFRT is superior or equivalent to high‐intensity training. These potential benefits may be related to local hypertrophy, systemic hemodynamic modulation, and neuroprotective signaling, although these mechanisms have not been fully validated in human stroke survivors. However, confidence remains limited, and methodological heterogeneity limits clinical generalization. Future research should prioritize protocol standardization, long‐term follow‐up, placebo‐controlled designs, and mechanistic validation in humans. Cognitive and upper‐limb outcomes remain underexplored. Only through such rigorous investigation can BFRT be integrated into evidence‐based stroke care.

## Author Contributions

Conceptualization: J.T., L.S. and J.J. Formal analysis: J.Z. and F.L. Writing – original draft preparation: J.T. T.H. and H.Y. Writing – review and editing. J.W. and J.W.Z. JT and LS contributed equally to this work as co‐first authors. JW and JZ are the co‐corresponding authors. All authors have read and agreed to the published version of the manuscript.

## Funding

This project was supported by the Research Project of Shanghai Second Rehabilitation Hospital (No. Y2025‐07).

## Ethics Statement

The authors have nothing to report.

## Conflicts of Interest

The authors declare that the research was conducted in the absence of any commercial or financial relationships that could be construed as a potential conflict of interest.

## Data Availability

The authors have nothing to report.
